# CORE-OM as a routine outcome measure for adolescents with emotional disorders: factor structure and psychometric properties

**DOI:** 10.1186/s40359-020-00459-5

**Published:** 2020-08-20

**Authors:** Veronica Lorentzen, Bjørn Helge Handegård, Connie Malén Moen, Kenth Solem, Kjersti Lillevoll, Ingunn Skre

**Affiliations:** 1 Department of Psychology, Faculty of Health Sciences, UiT The Arctic University of Norway, P.O. Box 6050 Langnes, N-9037 Tromsø, Norway; 2 Department of Child and Adolescent Mental Health, Divisions of Child and Adolescent Health, University Hospital of North Norway, 9038 Tromsø, Norway; 3 The Regional Centre for Child and Adolescent Mental Health – North, Faculty of Health Sciences, UiT The Arctic University of Norway, 9037 Tromsø, Norway; 4 The Norwegian Labour and Welfare Administration (NAV), Employment Advisory Services in Troms and Finnmark, 9811 Vadsø, Norway; 5 Substance use and Psychiatry unit, Department of Substance Use and Addiction Medicine, Clinic for Mental Health and Substance Use, Nordland Hospital, 8076 Bodø, Norway; 6 Clinic for Mental Health and Substance Use, University Hospital of North Norway, 9291 Tromsø, Norway

**Keywords:** Outcome measure, CORE-OM, Validation, Adolescents, Clinical, Emotional disorders

## Abstract

**Background:**

Instruments for monitoring the clinical status of adolescents with emotional problems are needed. The Clinical Outcomes in Routine Evaluation-Outcome Measure (CORE-OM) according to theory measures problems/symptoms, well-being, functioning and risk. Documentation of whether the theoretical factor structure for CORE-OM is applicable for adolescents is lacking.

**Methods:**

This study examined the factor structure and psychometric properties of the CORE-OM based on two samples of adolescents (age 14–18): youths seeking treatment for emotional problems (*N* = 140) and high school students (*N* = 531). A split half approach was chosen. An exploratory factor analysis (EFA) was performed on the first half of the stratified samples to establish the suitability of the model. A Confirmatory Factor Analysis (CFA) with the chosen model from the EFA was performed on the second half. Internal consistency and clinical cut-off scores of the CORE-OM were investigated.

**Results:**

The best fitting model only partially confirmed the theoretical model for the CORE-OM. The model consisted of five factors: 1) General problems, 2) risk to self, 3) positive resources 4) risk to others and 5) problems with others. The clinical cut-off score based on the all-item total was higher than in an adult sample. Both the all-item total and general problems cut-off scores showed gender differences.

**Conclusion:**

The factor analysis on CORE-OM for adolescents resulted in a five-factor solution, and opens up for new subscales concerning positive resources and problems with others. A 17-item solution for the general problems/symptoms scale is suggested. We advise developers of self-report instruments not to reverse items, if they do not intend to measure a separate factor, since these seem to affect the dimensionality of the scales. Comparing means for gender in non-clinical samples should not be done without modification of the general emotional problem and the positive resources scales. Slightly elevated CORE-OM scores (up to 1.3) in adolescents may be normal fluctuations.

## Background

Emotional disorders represent the most prevalent mental health problem in adolescence, and the comorbidity among emotional disorders is high. The onset of emotional problems typically occurs in childhood and adolescence or early adulthood [1]. There is a call for screening tools that can detect mental health problems in adolescents and determine their clinical status. Valid and reliable routine outcome measures are key tools in monitoring treatment effects and for detecting and preventing treatment failure [2]. There is a need for transdiagnostic measures that address comorbidity and are sensitive to change to monitor the treatment of adolescents.

The self-report Clinical Outcomes in Routine Evaluation-Outcome Measure (CORE-OM) is a 34-item questionnaire using a 5-point Likert scale from 0 (not at all) to 4 (most of the time). CORE-OM is widely used measure in outpatient mental health and counselling services and in psychotherapy research with adult patients [3–5]. The CORE-OM items were chosen based on their clinical significance and their sensitivity to change in psychological status [3, 4]. The CORE-OM theoretically covers four dimensions: Well-being (4 items); Functioning (12 items); Problems/symptoms (12 items); and Risk (6 items) [6, 7]. ‘Well-being’ refers to a patient’s sense of life quality and emotional health. ‘Problems/symptoms’ is associated with psychological health issues such as anxiety and depression symptoms, reactions to trauma, and physical complaints. ‘Functioning’ relates to interpersonal, social and general functioning in daily life. A high correlation has been found among these three domains [5, 8, 9], and combining them into a general psychological distress scale called All-items-minus Risk has been recommended [10]. ‘Risk/harm’ includes items covering harm to self and suicidal ideation (risk-to-self items) and violent behaviour and threats towards other people (risk-to-others items) [6, 11]. It has been recommended that risk be monitored separately to help the clinician detect a patient’s thoughts and plans regarding self-harm, suicide and violence [10].

The original CORE-OM [5] has been translated into more than 20 languages and has good psychometric properties in adult samples [7, 8, 12–15]. The CORE-OM has been benchmarked in student counselling and primary care service users aged 16 to 24 [16], however, this study lacked a control group of non-service users.

One version of the CORE developed for young people aged 11–16 is the CORE-YP [17]. However, the CORE-YP includes only ten of the CORE-OM items, phrased in simplified wording, and it is not adapted for the whole age span of adolescents (up to age 18) received in Norwegian child and adolescent mental health outpatient services. The CORE-OM, with its 34 items, gives more detailed information when needed; it addresses the most common comorbid symptoms in emotional disorders, is sensitive to change, and exists in a ten-item version (the CORE-10) that can be used session by session. Measures with these qualities are scarce in child and adolescent mental health services. For researchers and clinicians, it is more convenient to use one tool for adolescents, both for longitudinal research and to monitor treatment, without having to change tools due to changing age norms. In Norway youths up to age 18 are referred to child and adolescent mental health services, while individuals aged 18 and older are referred to adult outpatient services.

Since the CORE-OM addresses anxiety, depression, the aftermath of trauma, physical complaints, daily life functioning, subjective well-being, and risk to self and others, it is a highly relevant outcome measure, not only for adults but also for adolescent service users, and is particularly relevant for those with emotional problems. However, there is no existing validation of the full CORE-OM scale in high school age adolescent samples.

There is a need for knowledge about whether the test parameters for the CORE-OM in youths are comparable with those obtained in adult populations.

Previous research on the factor structure of the CORE-OM [9] has not fully confirmed the theoretically derived sub-scales but has rather indicated a structure constituted by a g-factor of psychological distress and residualized latent theoretically derived domains (Symptoms/Problems, Functioning and Well-being). ‘Risk-to-self’ correlates with the g-factor, while ‘risk-to-others’ has a poor fit in the structural models [9]. Several factor analytic evaluations of the CORE-OM have been performed. The test developers [5] suggested a first component that explained 38% of the variance, a risk component and a positively worded component. In later factor analyses some of the same researchers [9] suggested a bifactor model with an overall g-factor, a method factor (with positively and negatively keyed items) and risk to self and others. But, their best fitting model included the well-being, psychological problems, and functioning domains, although most of the factor loadings associated with these three domains were small after the g-factor was accounted for. In the Norwegian version [7] a bifactor model was also suggested, but here the method factor did not contribute to improve model fit. The model considered best in that study was a bifactor model with a general distress factor and the four CORE-OM domains. Investigations on the British version suggested using “Mokken Scaling” [10]. This scale is unidimensional resulting in a general distress factor, with items differentiating between more or less severe levels of stress. This approach result in the well-being items giving information about lower levels of stress, while the risk items covers higher levels of stress, with the other items in between. When the CORE-OM was constructed [5], the majority of items were statements about psychological distress or negative life situations, i.e., psychological distress. Most of the items were thus negatively keyed. However, eight items were positively keyed, allegedly to mitigate response bias [5]. However, mixing negatively and positively keyed items may add method effects that threaten the construct validity of an instrument. Whether the method effects of negatively and positively keyed items are caused by responder error [18], by response bias [19], or because positive and negative utterances actually measure different constructs are, however, unclear. Lyne et al. [9] demonstrated that the positively and negatively keyed items in the CORE-OM formed two separate method factors across the theoretically defined domains. In the validation of the CORE-OM in adult Norwegian samples, these method factors were also observed but were deemed negligible [7].

The CORE-OM has the potential to discriminate between non-clinical and clinical adult populations [5, 7, 8, 12, 13, 15]. The recommended cut-off score for the CORE-OM in the adult population is 1.0 [20].

Gender effects have been found for the All-items score, with women generally scoring higher than men, and consequently, gender-specific cut-off scores have been recommended by some authors [5, 13]. Other validations of the CORE-OM have not recommended separate cut-off scores based on gender, suggesting that gender effects were small and negligible [7, 12].

CORE-OM and Beck Depression Inventory (BDI) scores correlate in clinical samples [21]. Adolescents tend to have higher scores than adults on the BDI [22, 23]. Findings suggest that younger respondents generally score higher on the CORE-OM than older respondents [5, 7, 8, 14] and motivate the present authors to ask whether separate cut-off scores for adolescents are needed, as has been found for the BDI [22, 23].

Since no study has evaluated the factor structure of CORE-OM and the reliability of its factors in an adolescent population, there is a need for such studies. Since several studies have shown that girls score higher than boys do on emotional problems [24, 25] it is interesting to evaluate whether this also is observed when using the CORE-OM. To be able to do such a comparison, a requirement is that the factor structure for boys and girls are similar. In the factor analytic framework this can be done evaluating measurement invariance of the factors, and mean comparisons on gender require at least partial scalar invariance [26].

The aims of this paper were to study the psychometric properties of the Norwegian version of the CORE-OM in an adolescent clinical sample selected for emotional problems and a non-clinical sample by

(I) Investigating the factor structure of the Norwegian CORE-OM in adolescents aged 14 to 18 years, by establishing the suitability of the model by Exploratory Factor Analysis (EFA), and performing Confirmatory Factor Analysis (CFA) with the chosen model of the EFA.
(II) Evaluating the internal consistency on the scales from the chosen model.
(III) Performing a measurement invariance analysis for gender based on the factor structure suggested by the factor analysis.
(IV) Comparing factor means for boys and girls in the non-clinical sample, if at least partial scalar invariance under (III) is achieved.
(V) Calculating clinical cut-off scores.

## Methods

### Design

A between-subjects cross-sectional survey study was used to examine the psychometric properties of the CORE-OM in samples of Norwegian adolescents aged 14–18 years.

### Samples

Data were gathered from two separate samples: a non-clinical and a clinical sample. The non-clinical sample (*n* = 531) was recruited for the purpose of this paper from four junior high schools and four senior high schools in both urban and rural areas in North Norway. The schools were randomized and drawn, and data were collected until at least 65 participants from each class grade were included. In the non-clinical sample (age 14–18, M = 15.91, SD = 1.45), there were 273 (51.4%) boys and 258 (48.6%) girls. The response rate was between 71.4 and 83.3%. Although the sampling procedure was systematic, the non-clinical sample should be viewed as a convenience sample.

The clinical sample consisted of patients (*n* = 140) recruited at CAMHS (Child and Adolescent Mental Health Services) located in two North Norwegian towns and one community centre. In the CAMHS sample (age 14–17, M = 15.72, SD = 1.15), there were 13 boys (9.3%) and 127 girls (90.7%). The adolescents in this sample were enrolled as participants in a psychotherapy research project, the SMART study [27] (‘Evaluation of short-term treatment for adolescents with emotional disorders in five children and adolescent CAMHS—A randomized controlled trial’ (ClinicalTrials.gov Identifier: NCT02150265); Regional Committee for Medical and Health Research Ethics (REC North); Reference number 2011/1937). Data for the present study were collected at enrolment, before treatment.

### Inclusion/exclusion criteria

The inclusion criterion for the non-clinical sample was being a student at a junior or senior high school, while the exclusion criterion was that the adolescent could not read or write Norwegian fluently. The inclusion criteria in the clinical sample were (1) age between 14 and 17 years; (2) a probable diagnosis of an emotional disorder as indicated by a score of at least 6 on the Strengths and Difficulties Questionnaire (SDQ) screening tool; and (3) maintenance of a maximum waiting time for necessary medical care of 6 weeks given by Norwegian health authorities. The exclusion criteria were (1) a diagnosis of pervasive developmental disorder (PDD); (2) psychotic symptoms; (3) anxiolytic or anti-depressant medication effects during the treatment period; and (4) inability to speak the Norwegian language.

### Ethics and consent

The study was performed in compliance with the Helsinki Declaration for research on humans and was approved by the REC North (Reference number 2011/1937).

All participants participated and consented according to the regulations governing the research project, with written parental consent provided for those under age 16 (REC North, Reference number 2011/1937). High scores on symptom and risk items were addressed by the responsible therapist or counsellor.

### CORE-OM scoring

All participants completed the CORE-OM in Norwegian translation [7] on paper with a pen or pencil. All participants provided information regarding age and gender. The scoring procedure for both samples followed the guidelines for scoring from Barkham et al. [3].

### Statistical analyses

Descriptive data for each of the 34 items, separately for the clinical and non-clinical sample, have been provided as additional material.

The following procedure in evaluating the factor structure of the CORE-OM was performed: The sample was randomly split into two equally sized halves, stratified on sample (clinical/non-clinical) giving an equal proportion of cases from the clinical and non-clinical samples in each half. One of the sample halves was a training sample where an exploratory factor analysis (EFA) was done, and the other half was a testing sample where the chosen model from the EFA was tested using confirmatory factor analysis.

In the EFA, different solutions with varying number of factors was evaluated. Model fit information for each model, difference in model fit between subsequent factor solutions, and the meaningfulness of the Geomin rotated factor loadings was used to guide the choice of the number of factors.

The CFA was carried out on the chosen model from the EFA. The WLSMV estimator and delta parameterization was used [26]. Model fit for the CFA was evaluated by the Chi-square test, the chi-square to degrees of freedom ratio, the RMSEA [28] and the CFI [29]. A significant Chi-square test indicates significant model misfit, but the chi-square test is both a function of sample size and the amount of misfit so we rely mostly on the RMSEA and CFI for model fit evaluation. Models with a chi-square/df ratio < 2 [30]. RMSEA below 0.05 and a CFI above 0.95 are typically considered to be well-fitting [31].

With the WLSMV estimator and using the default missing data handling method in Mplus, pairwise deletion is used to handle the missing observations. This means that a pair of observations is used in computing a polychoric correlation if both observations in the pair are observed. Overall, the covariance coverage percentage of data for the CFA part of the sample was between 96.1 and 100%, and with the highest proportion of missing observations for items 19 and 20 where 2.4 and 2.1%, respectively, were missing.

Outliers was evaluated by Cook’s d in the CFA analysis. The Cook’s d computes, for each subject, the overall influence that the subject has on the parameter estimates estimated in the analysis. Additional analyses without the individuals with the highest Cook’s d values was performed, and there was noted a very small improvement in the fit indices in the CFA analysis when those cases were removed. Since the overall results and conclusions were not affected to a large degree by the outliers, our results were based on the whole sample.

Reliability was evaluated by computing McDonald’s Omega [32–34] for ordinal items, reporting a 95% confidence interval for the reliability parameter [35]. Omegas if item is deleted with 95% confidence intervals are also reported [36] We computed item to total (using the rest of the items) correlations by a procedure shown in Raykov & Marcoulides [37] computing polyserial correlations.

Measurement invariance for gender is necessary for gender mean comparisons on CORE-OM scales. Measurement invariance was evaluated for a configural and a scalar model for the non-clinical sample. The low number of clinical boys in our study made it impossible to assess measurement invariance for gender using the clinical sample. With ordinal indicators Muthen & Muthen [26] recommends that factor loadings and thresholds as a unity, so metric invariance holding only factor loadings invariant between genders were not carried out. We compared the difference in model fit between the configural model and the scalar model was tested using the DIFFTEST option in Mplus. Modification indices were assessed for partial scalar invariance if full scalar invariance was not achieved through the DIFFTEST. Even though the use of modification indices are controversial, it is often considered, and Muthen & Muthen [26] recommends that equality constraints for factor loadings and thresholds are relaxed in tandem. Following their recommendations, scale factors for items with freely estimated loadings and thresholds were fixed at one for identification purposes.

Mean gender differences on latent factors were evaluated in the final partial scalar model.

All factor analyses were performed in Mplus (version 7.4). Omega coefficients were computed within R using a procedure described by Peters [33].

The acceptability of the data was assessed by analysing missing data. Chi-squared tests were conducted to explore the relationship between the missing items and groups and missing items and gender. ANOVA was conducted to determine whether there was a significant relationship between the missing items and age.

Jacobson and Truax’s [24] formula was used to calculate the cut-off score for discriminating between a clinical and non-clinical sample:

$$ \frac{{\mathrm{mean}}_{\mathrm{clin}}{\mathrm{sd}}_{\mathrm{norm}}+{\mathrm{mean}}_{\mathrm{norm}}{\mathrm{sd}}_{\mathrm{clin}}}{{\mathrm{sd}}_{\mathrm{clin}}+{\mathrm{sd}}_{\mathrm{clin}}} $$

## Results

### Acceptability

A maximum omission of 10% of the items was used as an indication of acceptability for the CORE-OM. Nine participants (1.7%) with an omission rate greater than 10% were removed from the dataset: three from the junior high school sample and six from the senior high school sample.

### Exploratory factor analysis on the CORE-OM

EFAs with from one to six factors was performed. Factor solutions with one and two factors did not show good model fit. Three factors gave significantly better model fit than two factors, four factors gave significantly better model fit than three, five had significantly better model fit than four, and six was better than five according to a Chi-square difference test (see Table 1 for model fit for the 1 to 6-factor solutions). The factor loadings for the five-factor solution are shown in Table 2.

**Table 1:** Model fit information EFA

# of factors	Chi-square	df	RMSEA	CFI	SRMR	Difference Χ^2^(df)
1	1544.3*	527	0.076	0.942	0.078	–
2	1124.1*	494	0.062	0.964	0.063	2 vs. 1: Χ^2^(33) = 328.8*
3	817.8*	462	0.048	0.980	0.048	3 vs. 2: Χ^2^(32) = 257.0*
4	686.2*	431	0.042	0.985	0.040	4 vs. 3: Χ^2^(31) = 125.6*
5	562.3*	401	0.035	0.991	0.032	5 vs. 4: Χ^2^(30) = 112.9*
6	482.8*	372	0.030	0.994	0.027	6 vs. 5: Χ^2^(29) = 83.1*

* *p* < .0005

**Table 2:** Geomin rotated factor loadings for the five-factor solution (loadings above 0.30 are shown)

Item	1	2	Factor 3	4	5
1 I have felt terribly alone and isolated	**0.53**				
2 I have felt tense, anxious or nervous	**0.83**				
5 I have felt totally lacking in energy and enthusiasm	**0.49**				
8 I have been troubled by aches, pains or other physical problems	**0.79**			0.36	
10 Talking to people has felt too much for me	**0.58**				
11 Tension and anxiety have prevented me doing important things	**0.69**				
13 I have been disturbed by unwanted thoughts and feelings	**0.70**				
14 I have felt like crying	**0.66**				
15 I have felt panic or terror	**0.68**				
17 I have felt overwhelmed by my problems^a^	**0.67**				
18 I have difficulty getting to sleep or staying asleep	**0.75**				
20 My problems have been impossible to put to one side	**0.57**				
23 I have felt despairing or hopeless	**0.40**	0.35			
27 I have felt unhappy	**0.42**	0.42			
28 Unwanted images or memories have been distressing me	**0.46**				
29 I have been irritable when with other people	**0.35**				0.32
30 I have thought I am to blame for my problems and difficulties	**0.35**	0.33			
9 I have thought of hurting myself		**0.74**			
16 I made plans to end my life		**0.76**			
24 I have thought it would be better if I were dead		**0.85**			
34 I have hurt myself physically or taken dangerous risks with my health		**0.67**			
3 I have felt I have someone to turn to for support when needed			**0.51**		0.31
4 I have felt O.K. about myself			**0.51**		
7 I have felt able to cope when things go wrong			**0.51**		
12 I have been happy with the things I have done			**0.60**		
19 I have felt warmth and affection for someone	0.32		**0.67**		
21 I have been able to do most things I needed to			**0.58**		
31 I have felt optimistic about my future^a^			**0.71**		
32 I have achieved the things I wanted to			**0.65**		
6 I have been physically violent to others				**0.69**	
22 I have threatened or intimidated another person				**0.78**	
25 I have felt criticised by other people					**0.67**
26 I have thought I have no friends					**0.45**
33 I have felt humiliated or shamed by other people					**0.87**

^a^ Items 17 and 31 have switched place in the English and Norwegian version of the CORE-OM

The five-factor solution was chosen, since the six-factor solution did not seem to add anything of substance. In Table 2 the factor loadings above 0.30 for each item are shown in bold for the five-factor solution. Factor 1 is interpreted as a general problem factor. All items for this factor are negatively keyed, and is a mix of what the manual describes as symptoms of anxiety, depression, physical problems, trauma, functioning and subjective well-being. Factor 2 consists of four risk-to-self items, i.e. four of the six “risk” items load highest on this factor. In addition, items 23, 27 and 30 loaded nearly as high on this factor as on Factor 1. These items points to symptoms of depression. One option is to remove such cross-loading items, but it seems important to include questions about unhappiness and despair in a questionnaire measuring symptoms of depression or general emotional problems. The level of risk of self-harm and general emotional problems are likely correlated, and it is therefore natural to observe some cross-loadings for these items.

The eight items loading highest on the third factor were all positively keyed, so this latent variable can be interpreted as positive resources that the adolescent possesses.

Factor 4 has two high-loading items that are interpreted as risk-to-others items.

For the fifth factor, items 25, 26 and 33 are functioning items related to functioning/relations with other people. Item 31 and item 3 had a cross-loading on this factor that was nearly as high as the loading on the general factor. Both these items are related to relations to other people, but the positive framing of item 3 give a higher loading on the positive resources latent variable, while item 31 (irritability with others) can both be a result of problems with others but also something that is related to general problems.

This factor structure gave the most interpretable factors in our opinion. Even though we got a significantly better fit with six factors compared to five, the sixth factor loads highest only on item 8. This item has to do with physical problems or pain and is possibly related to somatic symptoms, which is not asked for in detail in CORE-OM. The six-factor solution is not shown here, but with very few exceptions, the items were loading on the same factors as in the five-factor solution.

The two-factor and three-factor solutions gave a general factor plus additional factors that were hard to interpret. The four-factor solution had a general factor, Risk to others, Positive items and Problems with others as possible interpretations, and the difference between this and the five-factor solution was mainly that the risk to self-items loaded on the general problems factor.

### Confirmatory factor analysis

A CFA was performed on the five-factor model that was selected from the EFA, using the other half of the sample.

**Fig. 1:**
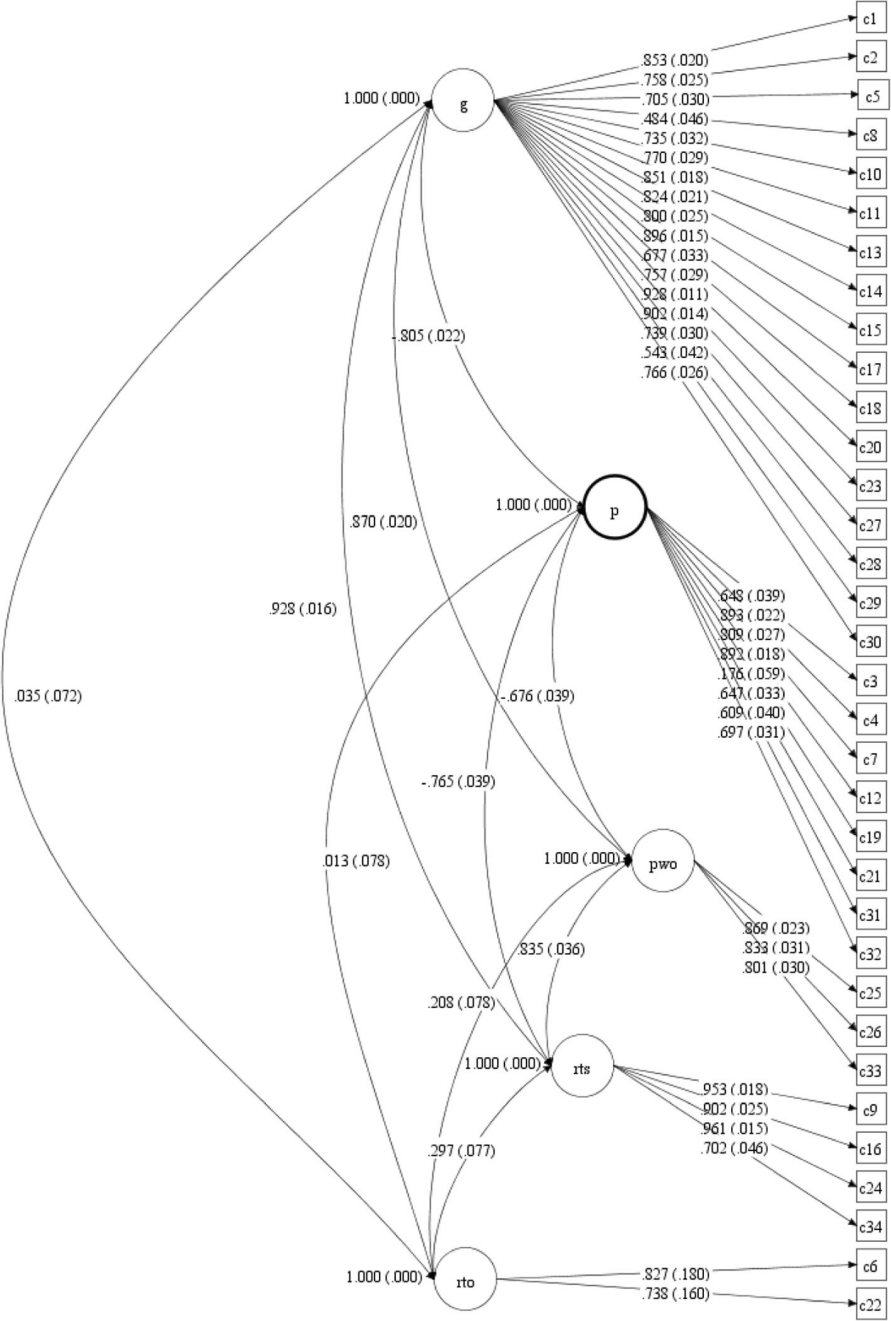
Confirmatory factor analysis of the CORE-OM. Five-factor standardized solution with standard errors. Factor correlations (with SE) are shown next to curved arrows. Latent measurement errors for each item are not shown in the figure. g = General problems; p = Positive resources; pwo = Problems with others; rts = Risk to others; rto = Risk to others

In this analysis, all factor loadings were significantly different from zero (see Fig. 1). Item 19 showed a loading that indicated a low association to the Positive resources latent variable.

Model fit information for this analysis showed a significant χ^2^(517) = 956.7 (*p* < .0005), and χ^2^/df = 1.85; RMSEA = 0.05 (90% CI: (0.045, 0.055)); CFI = 0.978. According to commonly used criteria for a “good” model, both the RMSEA and the CFI satisfy these benchmarks, as does the χ^2^/df ratio (< 2).

The estimated correlations between the latent variables showed high correlations among all factors except for the Risk-to-others latent variable.

### Reliability evaluation for the five factors: symptoms and problems, positive resources, risk to self, risk to others, problems with others

The CORE-OM items are measured on an ordinal (5-point Likert) scale. Therefore, we computed internal consistency reliability using the ordinal Omega coefficient [33]. Omega coefficients based on the CFA sample are shown in Table 3. The omega if item deleted and item to total correlations are shown in Table 4.

**Table 3:** Omega coefficients based on the CFA sample

Scale	Ordinal Omega	95% confidence interval
General problems	.958	(.952, .965)
Positive resources	.881	(.861, .901)
Problems with others	.862	(.836, .887)
Risk to self	.931	(.918, .943)
Risk to others	.576^a^	–

^a^ Spearman-Brown coeff

**Table 4:** Omega if item deleted and item to total correlations

	Omega if item deleted	95% CI^a^	Item to rest correlation^b^
General problems			
Item 1	.955	(.947, .962)	.783
Item 2	.956	(.949, .963)	.721
Item 5	.957	(.950, .964)	.668
Item 8	.960	(.953, .966)	.489
Item 10	.957	(.950, .964)	.652
Item 11	.956	(.949, .963)	.699
Item 13	.954	(.947, .962)	.807
Item 14	.955	(.947, .962)	.780
Item 15	.955	(.948, .962)	.759
Item 17	.954	(.946, .961)	.844
Item 18	.957	(.950, .964)	.637
Item 20	.956	(.949, .963)	.723
Item 23	.954	(.946, .961)	.849
Item 27	.954	(.947, .962)	.825
Item 28	.956	(.949, .963)	.692
Item 29	.959	(.953, .966)	.518
Item 30	.956	(.949, .963)	.705
Positive resources			
Item 3	.869	(.847, .891)	.614
Item 4	.859	(.835, .882)	.692
Item 7	.856	(.832, .880)	.715
Item 12	.848	(.823, .874)	.792
Item 19	.897	(.879, .914)	.288
Item 21	.870	(.849, .892)	.605
Item 31	.867	(.845, .890)	.621
Item 32	.863	(.840, .886)	.670
Risk Self			
Item 9	.896	(.876, .915)	.698
Item 16	.901	(.883, .920)	.723
Item 24	.909	(.892, .926)	.662
Item 34	.932	(.919, .945)	.575
PWO^c^			
Item 25	–	–	.726
Item 26	–	–	.639
Item 33	–	–	.648
Risk Others			
Item 6	–	–	.610^d^
Item 22	–	–	

^a^ See Dunn, Baguley & Brunsden [35]; ^b^Polyserial correlations between the item and the total (without the item) of the scale; ^c^Problems with others; ^d^ Polychoric correlation

Deleting one item from the scale, produces a small decrease in the reliability score for most of the items. For the general problem scale, reliability is not affected much by deletion of a single item, partly because of the large number of items in this scale. Reliability is maximized if item 8 (“I have been troubled by aches, pains or other physical problems “) is deleted with an increase from .958 to .960. Also, for item 29 there is an increase in Omega if the item is deleted. For the positive scale, item 19 (“I have felt warmth and affection for someone “) performs worst. Dropping this item increases the Omega reliability score from .881 to .897. For the Risk to self-scale, deleting item 34 (“I have hurt myself physically or taken dangerous risks with my health“) increase the Omega coefficient slightly.

The item to total correlations gives similar results as the Omega if item deleted, with the same items as mentioned above associated with the lowest item-total correlations. Particularly item 19 may be problematic to include as an indicator of the positive resources latent variable.

### Measurement invariance for gender on the non-clinical sample

The configural model that allows all parameters to be estimated freely for the genders showed good model fit (χ^2^(1034) = 1610.4 (*p* < .0005), RMSEA = 0.046 (90% CI: (0.041, 0.050)), CFI = 0.969). Reasonable model fit for the configural model is necessary for further measurement invariance testing. The scalar model holding factor loadings and thresholds invariant across the genders had model fit: χ^2^(1153) = 1751.9 (*p* < .0005), RMSEA = 0.044 (90% CI: (0.040, 0.048)), CFI = 0.968. The DIFFTEST in Mplus showed significantly worse model fit for the scalar model compared with the configural model (χ^2^(119) = 207.1; *p* < 0.0005), indicating that holding all loadings and thresholds to be equal across genders are not warranted, so the requirement of full scalar invariance does not hold. Similar CFI and RMSEA values for the configural and scalar model could indicate that there are problems with a relatively few loadings and thresholds. Partial scalar invariance was therefore evaluated using modification indices.

The largest modification index for the scalar model was associated with the lowest threshold for item 14 (“I have felt like crying”). This is the item with a large difference in the distribution for boys and girls. Relaxing the loading and the threshold constraints for this item made model fit slightly better, but still the DIFFTEST was significant: χ^2^(115) = 174.1 (*p* < 0.0005). Next, we relaxed the loading and thresholds for item 29, and the DIFFTEST was still significant: χ^2^(111) = 161.5 (*p* < 0.0005).

We did modify the scalar model by relaxing the constraints one by one according to the largest modification index. Table 5 shows the steps taken in this process:

**Table 5:** Steps in showing partial scalar invariance

Step	Largest modification index	Chi-square (df)	RMSEA (90% CI)	CFI	DIFFTEST (Configural vs. partial scalar)
A	Item 14^a^ Threshold 1	1713.8 (1149)***	0.043 (0.039, 0,047)	0.970	∆χ^2^(115) = 174.1***
B	Item 29^b^ Loading	1702.2 (1145)***	0.043 (0.038, 0,047)	0.970	∆χ^2^(111) = 161.5***
C	Item 4^c^ Threshold 3	1679.3 (1141)***	0.042 (0.038, 0,046)	0.971	∆χ^2^(107) = 140.7* *p* = .016
D	Item 31^d^ Loading	1665.9 (1137)***	0.042 (0.037, 0,046)	0.972	∆χ^2^(103) = 127.3, *p* = .053 n.s.
E	Item 19^e^ Threshold 4	1662.0 (1133)***	0.042 (0.038, 0,046)	0.972	∆χ^2^(99) = 117.8, *p* = .10 n.s.

* *p* < .05, *** *p* < .0005. *n.s.* non-significant. ^a^ I have felt like crying; ^b^ I have been irritable when with other people; ^c^ I have felt O.K. about myself; ^d^ I have felt overwhelmed by my problems; ^e^ I have felt warmth and affection for someone

The items that the data indicate might be most problematic to establish scalar invariance are shown in Table 5. Item 14 has very different distribution for boys and girls. Maybe including an item about crying is not a good idea when assessing differences on emotional problems for boys and girls. Admitting to crying is probably very different for the genders, and a boy and a girl with the same amount of problems might answer this question very differently (gender specific differences). Item 4 has the same tendency as for item 14 with a much higher proportion of boys reporting satisfaction with themselves than girls. Items 19 and 29 had the lowest standardized loadings both for boys and girls.

### Gender differences on the latent factors

Based on the final partial scalar model (E), there was a significant gender difference in the factor means for the General problems latent variable (z = − 7.52; *p* < .0005; boys scoring lower than girls), Positive resources (z = 5.46; *p* < .0005; boys scoring higher than girls), Problems with others (z = − 5.02; *p* < .0005; boys scoring lower than girls), and Risk-to-others (z = 3.11; *p* = .002; boys scoring higher than girls). There were no significant differences between boys and girls on the latent Risk-to-self variable.

### Clinical cut-off score

Clinical cut-off scores were estimated by employing Jacobson and Truax’ [38] formula.

Before calculating the cut-off score, participants in the non-clinical sample that reported being in treatment were excluded (*n* = 23). The estimated CORE-OM all-items cut-off score according to Jacobson and Truax’ formula was 1.31 (girls: 1.44; boys: 1.02). For the 17-item general distress scale the cut-off was 1.51 (girls: 1.69; boys: 1.09).

## Discussion

The main findings in this validation of the CORE-OM in a mid-adolescent sample were a new factor solution and a higher cut-off score than reported in adult samples. The EFA resulted in a five factor solution, and the factor contents were interpreted as general problems, positive resources, risk to self, risk to others, and problems with others. The CFA model fit for this model was good. The measurement invariance analysis for gender should not be performed without modification of the scale. The clinical cut-off score based on the all-item total was higher than in an adult sample. Both the all item total and general problems cut-off score showed gender difference.

### Factor analysis and reliability

From the exploratory factor analysis, based on the training part of the training sample, a five-factor model was interpreted to be the best candidate for model evaluation. In the EFA, this model had improved model fit over factor solutions with less factors, and had factors that were interpreted as General problems, Positive resources, Risk to self, Risk to others and Problems with others. In the following confirmatory factor analysis, done on the testing part of the sample, model fit for this model can be characterized as good.

The developers of the CORE-OM manual describes the instrument as a four-dimensional measure with dimensions of Subjective well-being, Problems, Functioning and Risk [6]. There may be many reasons why data from the present youth sample yielded a different factor structure. We believe that one main reason for this is that eight of the 34 items are positively keyed. Lyne et al. (2006) [9] showed that for CORE-OM on an adult sample, method factors related to positive and negative wording of the items played a role in achieving acceptable model fit. In our sample, all the eight positively keyed items loaded on the same factor in the five-factor EFA. It seems that when the adolescents answer these items, the positive resources in their lives are prompted, rather than just negative aspects. This highlights that assuming that a low score on a positively keyed item reflects the same as a high score on a negatively keyed item is problematic. According to the tripartite theory of anxiety and depression [39] negative affect and lack of positive affect may represent separate dimensions of internalizing problems. The current factor solution supports that negative affect and lack of positive affect are not two sides of the same coin.

Combining all the positively keyed items to a separate subscale not only solves the problem of reversed items, but also produces a substantially easier subscale to interpret than the theoretically derived Well-being scale, since it reflects resources, wellbeing and self-efficacy.

Incidentially, one of the positively keyed items had a much lower factor loading in the CFA than the other items. Although item 19 (“I have felt warmth and affection for someone») is positively keyed it differs in content from the rest of the positive items. This item may measure traits like empathy or affection directed towards other people, and not necessarily positive feelings about themselves. Also, removing this item from the scale improves the Omega reliability score by nearly 0.02, and this item had an item to rest-correlation below 0.30. One other reason for the low factor loading for item 19 may be that the Norwegian translation of the word “affection” is a word that is probably not used among Norwegian adolescents nowadays. Thus, a revision of the Norwegian translation is recommended.

The risk items split into two distinct factors. The risk to others items (item 6 and 22) correlates highly with each other but little with the other items in the questionnaire. We also see this through low factor correlations between the latent Risk-to-others variable and the other four latent variables in the CFA. In the EFA, a Risk to other scale shows up early, although it is questionable whether these two items cover a large enough range of such a dimension.

The Risk to self-dimension seems more robust, having a high reliability score for the internal consistency. The factor correlation between Risk-to-self and General problems is very high (> 0.90), and this seems natural as having many symptoms of problems may impact self-harm and suicidal ideation. Cross-loadings between Risk-to-self and General problems were evident for the items “I have felt despairing or hopeless” and “I have felt unhappy”. Although such items may indirectly indicate risk of self-harm, we believe that these items are more direct indicators of the severity of of emotional problems.

The reliability analysis revealed that the Omega would increase slightly if item 34 “I have hurt myself physically or taken dangerous risks with my health” was removed from the scale. This item may or may not be related to intensions of self-mutilations or suicide. The other items within the Risk to self-scale are more directly associated with such intentions, while taking dangerous risks may be sensation-seeking behavior not directly associated with self-harm intentions.

For the General problems scale, half (17) of the CORE-OM items loaded highest on this variable in the EFA. For this scale, the Omega reliability would improve slightly if the items 8 and 29 were removed from the scale, and these two items had the lowest item to rest correlations for the items within the General problems scale. Item 8 (“I have been troubled by aches, pains or other physical problems “) may be caused by mental health issues but can also be a result of injuries, physical disease and other issues not related to emotional problems. Increased reliability removing this item from the general problem scale may be an indication of this. For item 29 (“I have been irritable when with other people”) was probably the item that was most difficult to place. It loaded moderately on the General problem latent variable, and cross-loaded on the Problems with others latent variable. To be irritable when with others can be an indicator of problems with the functioning with others, but can also be an indicator of emotional problems since irritability may be associated with several traits or conditions [40].

Finally, items 25, 26 and 33 loaded on the Problems with others factor. These items have to do with relationships with others. Lyne et al. [9] pointed at the same three items as belonging to a common factor. After accounting for a general distress factor, these three items were the only items that had meaningful loadings on their residualized Functioning factor. This highlights that feelings of humiliation or critique from others and having no friends may form a separate factor in the CORE-OM instrument.

### Measurement invariance for gender

We did a measurement invariance analysis for gender, to evaluate whether it is reasonable to make mean comparisons between girls and boys using CORE-OM.

Comparing the configural model and full scalar model, we found that scalar model fit significantly worse than the configural model, and this indicates that one cannot compare means for boys and girls without modifications to the scales. After 4–5 steps of relaxing constraints in the scalar model, we found a partial scalar model that did not fit significantly worse than the configural model. In comparing means for boys and girls on the CORE-OM scales, one should probably be careful in using the items 14, 29, 4, 31 and 19.

Different researchers rely on different fit statistics when evaluating measurement invariance Putnick and Bornstein [41] show that many consider that a small change in CFI or RMSEA going from a configural to a scalar model could indicate scalar invariance. The change in CFI and RMSEA shown for gender invariance in the non-clinical sample in the present study, is very small, and within the limits of full scalar invariance mentioned by Putnick and Bornstein [41]. However, it is problematic if one chooses the change in χ^2^ as criterion for invariance when it is non-significant and other criteria when it is significant. We used a data driven method (modification indices) instead to establish partial scalar invariance. Partial scalar invariance can be concluded when a large majority of the items on the factors is invariant [42] The use of modification indices is also controversial [41], but can be helpful in determining items that are problematic. For example, our analysis showed that item 14 in CORE-OM (“I have felt like crying”) may be an item that is problematic to include when symptoms of depression or anxiety are to be compared between the genders. Boys and girls report very differently on this item, and this difference cannot be attributed only to the amount of emotional problems on the latent scale the adolescents have but also to some gender specific traits.

#### Gender differences in the non-clinical sample

We compared factor means for male and female adolescents in the non-clinical group using the final partial scalar model. Boys and girls differed on four of the five latent variables. A non-significant difference between the genders was found for the risk to self-variable. For the non-clinical group few adolescents had thoughts of self-harm. The girls scored higher than boys did on the general factor, and that has also been shown in other studies [5, 13]. Boys scored significantly higher on the risk to others factor. This is consistent with other validations of the scale [7, 14] .

For the positive resources latent variable, girls scored significantly lower than boys. Finally, for the Problems with others factor the girls scored higher than boys. The items in this scale have to do with feelings of having been criticized, humiliated, made shameful or having no friends, and are as such about emotional relations with others. Girls tend to use emotional coping skills more often than boys, and help from others, while boys tend to devaluate such emotional expressions [43], hence stronger feelings related to emotional relationships can be the result. In the Japanese version of CORE-OM the female participants showed lower scores on “close relationships” subscales [14].

Factor correlations between the latent variables in the chosen factor solution were high, except for those involving risk to others. Similar gender differences for general emotional problems, positive resources and problems with others can be a sign that related concepts are being involved.

### Mixing positive and negative items in a questionnaire

Lyne et al. [9] concluded their article, studying 2140 adult patients, that the most useful scoring method of the CORE-OM would be to compute a general total score based on the 28 non-risk items and a risk total based on the remaining six items. The main difference between the 17-item general problems scale from the present study and the 28-item non-risk scale is the exclusion of the positively keyed items from the 17-item version.

One of the reasons for including both positively and negatively keyed items in a questionnaire is to reduce acquiescence bias (response style bias, respondents tending to agree with statements) [44]. However, positively and negatively keyed items may involve different cognitive processes [45, 46] and this is one of the reasons that a positive item latent variable showed up in the EFA. It is a paradox that including some positively keyed items in a questionnaire consisting mostly of negatively keyed items, in order to mitigate acquiescence bias, seems to confuse the responders and therefore makes the instrument less valid and scales less reliable.

### Clinical cut-off score

The original validation of the CORE suggested a clinical cut-off of 1.2 [5], and later validations have suggested a cut-off point as low as 1.0 [47] to define clinical caseness. However, in these adolescent samples, the cut-off score on the All-items CORE-OM was 1.31, 1.44 (girls) and 1.02 (boys). This finding needs to be replicated, but it corresponds well with the finding that youths also score higher than adults on the BDI [22, 23]. Consistent of the results from the present study we also recommend the 17-item factor as a measure of general problems. The positively keyed items do not interfere with this factor and the problem with others items are also excluded. In this way we have a more reliable measure on emotional problems and the cut-off scores for this factor is suggested as an alternative to the established All items minus Risk score. The rationale for this is that the All items minus Risk 28-item score includes all reversed items, and may thus actually underestimate the level of emotional distress experienced by patients. The cut-off scores for both All-items and the 17-items general distress factor show gender differences, with girls scoring higher than boys and a higher score than in adult samples [7]. We suggest that the cut-off scores either is gender specific or that the cut-off for gender combined is set lower to accommodate for the boys lower scoring, as suggested by Connell et al. [47].

### Limitations

The clinical sample may not be representative of the entire CAMHS population due to the sample being preselected based on symptoms of emotional problems. Furthermore, patients evaluated as suicidal were excluded from the sample because they could not be subjected to the 6-week waiting condition. However, since the CORE-OM was mainly developed to monitor outpatient treatment and is not the outcome measure of choice for psychosis or conduct disorder, the present clinical sample probably has a high density of the phenomena that the CORE-OM was designed to monitor.

The age span in the non-clinical sample was 14–18, while the age range in the clinical sample was 14–17. The reason for this is that Norwegian CAMHS receives only those younger than age 18 as patients, while youths 18 and older are referred to mental health services for the adult population. However, in high school, enrolment in different grades is based on the year of birth. We decided not to exclude the 18-year-olds from the non-clinical sample. Furthermore, the mean age in the two samples is similar.

Due to the low rate of males in the clinical sample, the mean and standard deviation in the male clinical sample used in the Jacobson and Truax formula have large standard errors. Therefore, the clinical cut-offs for boys are encumbered with uncertainty.

## Conclusions

Although the present version of CORE-OM shows promising psychometric properties, there are some challenges with the instrument. Leaning on van Sonderen et al. [48] and Suárez-Alvarez et al. [44], we believe that using a mix of positively and negatively keyed items should be avoided, if the intention is not to measure separate dimensions. However, the five-factor solution found in this validation both had a good model fit, and not the least, yielded clinically meaningful subscales. According to the factors found in this study we recommend the 17-item factor as a more reliable measure of general problems. Comparing means for gender in non-clinical samples should not be done without modification of the general emotional problem and the positive resources scales. This should be objectives for future revisions of the scale.

## Supplementary information

**Additional file 1.**

## Data Availability

The datasets generated and analysed during the current study and the full study protocol are available from the corresponding author on reasonable request.

## Abbreviations

CORE-OM: Clinical Outcome in Routine Evaluation-Outcome Measure
EFA: Exploratory Factor Analysis
CFA: Confirmatory Factor Analysis
BDI: Becks Depression Inventory
SDQ: Strengths and Difficulties Questionnaire
CAMHS: Child and Adolescent Mental Health Services
ANOVA: Analysis of Variance
WLSMV: Weighted Least Squares Means and Variance adjusted
RMSEA: Root Mean Square Residual
CFI: Comparative Fit Index
