# Supplementary material for: CORE-OM as a routine outcome measure for adolescents with emotional disorders: factor structure and psychometric properties
Source: BMC Psychol. 2020 Aug 20;8:86. doi: 10.1186/s40359-020-00459-5 (PMC7439542; doi:10.1186/s40359-020-00459-5)
Supplement: Supplementary file 1 — Additional file 1. [file 40359_2020_459_MOESM1_ESM.pdf]

Additional File 1. Descriptive statistics for the 34 CORE-OM items for the non-clinical and clinical sample.

	Non-clinical sample					Clinical sample				
Item	M[a] (SD[b])	50p[c]	IQR[d]	S[e]	K[f]	M (SD)	50p	IQR	S	K
1 I have felt terribly alone and isolated	0.76 (0.94)	0	1	1.12	0.62	1.81 (1.21)	2	2	–0.03	–0.81
2 I have felt tense, anxious or nervous	1.27 (1.07)	1	2	0.55	–0.42	2.44 (0.97)	3	1	–0.25	–0.63
3 I have felt I have someone to turn to for support when needed	3.05 (1.13)	3	2	–1.13	0.49	2.35 (1.20)	2	2	–0.19	–0.91
4 I have felt O.K. about myself	2.58 (1.13)	3	1	–0.44	–0.65	1.21 (0.98)	1	2	0.49	–0.32
5 I have felt totally lacking in energy and enthusiasm	1.64 (1.05)	2	1	0.18	–0.52	2.73 (1.05)	3	2	–0.60	–0.25
6 I have been physically violent to others	0.26 (0.61)	0	0	3.11	12.33	0.16 (0.51)	0	0	3.80	15.45
7 I have felt able to cope when things go wrong	2.99 (0.96)	3	2	–0.72	–0.05	1.82 (1.04)	2	1	0.29	–0.17
8 I have been troubled by aches, pains or other physical problems	1.30 (1.15)	1	2	0.59	–0.53	2.01 (1.35)	2	2	–0.01	–1.13
9 I have thought of hurting myself	0.31 (0.79)	0	0	2.73	6.99	1.32 (1.34)	1	2	0.55	–0.97
10 Talking to people has felt too much for me	0.74 (1.01)	0	1	1.32	1.01	1.70 (1.08)	2	1	0.07	–0.42
11 Tension and anxiety have prevented me doing important things	0.64 (0.99)	0	1	1.50	1.41	1.75 (1.23)	2	2	0.07	–0.91
12 I have been happy with the things I have done	2.68 (0.98)	3	1	–0.50	–0.12	1.61 (0.89)	2	1	0.29	0.20
13 I have been disturbed by unwanted thoughts and feelings	1.17 (1.16)	1	2	0.79	–0.22	2.56 (1.12)	3	1	–0.43	–0.60
14 I have felt like crying	1.26 (1.25)	1	2	0.70	–0.54	2.65 (1.11)	3	1	–0.77	0.07
15 I have felt panic or terror	0.65 (0.91)	0	1	1.37	1.29	1.68 (1.13)	2	1	0.20	–0.72
16 I made plans to end my life	0.17 (0.55)	0	0	3.86	16.62	0.49 (0.89)	0	1	1.82	2.22
17 I have felt overwhelmed by my problems[a]	0.80 (1.05)	0	1	1.14	0.33	2.37 (1.20)	2	1	–0.27	–0.82
18 I have difficulty getting to sleep or staying asleep	1.22 (1.24)	1	2	0.71	–0.56	2.36 (1.36)	3	2	–0.46	–0.99
19 I have felt warmth and affection for someone	2.43 (1.26)	3	1	–0.45	–0.79	2.24 (0.98)	2	1	–0.03	–0.58
20 My problems have been impossible to put to one side	1.12 (1.20)	1	2	0.87	–0.27	2.34 (1.08)	2	1	–0.43	–0.32
21 I have been able to do most things I needed to	2.88 (1.08)	3	2	–0.90	0.21	2.14 (0.96)	2	2	0.02	–0.32
22 I have threatened or intimidated another person	0.24 (0.54)	0	0	2.80	10.29	0.12 (0.47)	0	0	4.13	17.16
23 I have felt despairing or hopeless	0.83 (1.04)	0	1	1.12	0.40	2.10 (1.24)	2	2	–0.17	–0.97
24 I have thought it would be better if I were dead	0.38 (0.85)	0	0	2.48	5.76	1.36 (1.42)	1	2	0.63	–0.96
25 I have felt criticised by other people	1.04 (1.09)	1	2	0.86	–0.06	1.95 (1.34)	2	2	–0.07	–1.19
26 I have thought I have no friends	0.50 (0.89)	0	1	1.97	3.51	1.44 (1.35)	1	2	0.60	–0.81
27 I have felt unhappy	0.92 (1.09)	1	2	1.07	0.35	2.34 (1.28)	3	2	–0.40	–0.93
28 Unwanted images or memories have been distressing me	0.82 (1.09)	0	1	1.31	0.94	1.67 (1.34)	2	3	0.26	–1.08
29 I have been irritable when with other people	0.88 (0.99)	1	1	1.06	0.63	1.79 (1.20)	2	2	–0.01	–0.82

30 I have thought I am to blame for my problems and difficulties	1.25 (1.25)	1	2	0.73	–0.48	2.30 (1.23)	2	2	–0.33	–0.78
31 I have felt optimistic about my future[a]	2.38 (1.31)	3	2	–0.39	–0.93	1.61 (1.08)	2	1	0.42	–0.31
32 I have achieved the things I wanted to	2.57 (0.98)	3	1	–0.46	–0.08	1.82 (0.87)	2	1	0.03	0.16
33 I have felt humiliated or shamed by other people	0.67 (0.92)	0	1	1.43	1.70	1.01 (1.06)	1	2	0.73	–0.56
34 I have hurt myself physically or taken dangerous risks with my health	0.37 (0.85)	0	0	2.52	6.01	0.63 (1.08)	0	1	1.56	1.30

a. M = Mean; b. SD = Standard deviation; c. Median; d. Inter-quartile range; e. Skewness; f. Kurtosis. The numbers in this table have been computed in IBM SPSS, version 26.
